# Understanding the burden experienced by patients with mild cognitive impairment due to Alzheimer's disease and dementia due to Alzheimer's disease at different stages of disease severity in a real‐world setting

**DOI:** 10.1002/alz70858_097342

**Published:** 2025-12-24

**Authors:** Riya Arora, Niels Juul Brogaard, Kristian Steen Frederiksen, Nikolaos Scarmeas, Sarah Cotton, Chloe Walker

**Affiliations:** ^1^ Novo Nordisk Service Centre India Pvt Ltd, Bangalore, India; ^2^ Novo Nordisk A/S, Søborg, Denmark; ^3^ Danish Dementia Research Centre, Dept. of Neurology, Copenhagen University Hospital ‐ Rigshospitalet, Copenhagen, Denmark; ^4^ Department of Clinical Medicine, Faculty of Health and Medical Sciences, University of Copenhagen, Copenhagen, Denmark; ^5^ 1st Department of Neurology, Medical School, National and Kapodistrian University of Athens, Athens, Greece; ^6^ Department of Neurology, Columbia University, New York, NY, USA; ^7^ Adelphi Real World, Bollington, United Kingdom

## Abstract

**Background:**

People with Alzheimer's Disease (AD) inevitably progress from preclinical stage, to experiencing mild cognitive impairment (MCI) to dementia. Our aim was to characterise the burden experienced by patients, delineated by disease severity.

**Method:**

Data were drawn from the Adelphi Real World AD Disease Specific Programme™, a cross‐sectional survey of physicians and their patients with MCI due to AD or dementia due to AD (clinically diagnosed or biomarker confirmed) in Canada, France, Germany, Italy, Spain, the United Kingdom, Japan and the United States between December 2022 – March 2024. Physicians were asked to report data on demographics and symptoms for their next nine consecutively consulting patients, followed by one patient with a biomarker‐confirmed MCI due to AD diagnosis. Patients self‐reported the impact of disease on their lifestyle and responses were grouped by theme. Data was analysed by disease severity according to the patient's current Mini‐Mental State Examination (MMSE) score. Analyses were descriptive.

**Result:**

Overall, 829 physicians reported data on 4202 patients (10.3% with an MMSE of 26‐30, 37.8% 21‐25, 46.7% 11‐20, 5.3% 0‐10). In total, 558 patients with an MMSE between 11‐30 self‐reported data. Overall, patients had a mean (standard deviation) age of 75.5 (8.2) years and 50.3% were male. Physicians reported that functional impairment of activities were experienced by 34.6%, 62.2%, 81.0% and 90.1% of patients with an MMSE of 26‐30, 21‐25, 11‐20 and 0‐10, respectively; and behavioural symptoms by 44.5%, 65.3%, 77.3% and 86.1% of patients. Most frequently experienced symptoms included impairment of short‐term memory (86.1% of patients with an MMSE of 26‐30; 85.4% 21‐25; 87.8% 11‐20; 95.5% 0‐10), difficulties in concentration (36.9%; 47.6%; 56.2%; 74.9%) and difficulty recalling names/words (35.0%; 41.4%; 52.5%; 66.8%).

Patients reported experiencing greater isolation (54.2% of patients with an MMSE of 26‐30; 62.0% 21‐25; 74.5% 11‐20) and lower self‐esteem/confidence (55.9%; 63.7%; 54.5%) since receiving their diagnosis.

**Conclusion:**

Impairment across cognitive, functional and behavioural symptoms/abilities are experienced by patients with MCI due to AD and dementia due to AD, irrespective of disease severity. Patients report isolation and feelings of lower self‐esteem after diagnosis. Early intervention to slow disease progression is important to decrease patient burden.

